# Chenodeoxycholic Acid Derivative HS-1200 Inhibits Hepatocarcinogenesis and Improves Liver Function in Diethylnitrosamine-Exposed Rats by Downregulating MTH1

**DOI:** 10.1155/2017/1465912

**Published:** 2017-02-05

**Authors:** Miao Xu, Qi Zhao, Donghui Shao, Hui Liu, Jianni Qi, Chengyong Qin

**Affiliations:** ^1^Department of Gastroenterology, Shandong Provincial Hospital Affiliated to Shandong University, Jinan, Shandong 250021, China; ^2^Department of Gastroenterology, Jinan Hospital, Jinan, Shandong 250013, China; ^3^Central Laboratory, Shandong Provincial Hospital Affiliated to Shandong University, Jinan, Shandong 250021, China

## Abstract

*Aim*. To investigate the effects of HS-1200 on liver tumorigenesis and liver function in a diethylnitrosamine- (DEN-) induced hepatocellular carcinoma (HCC) rat model.* Methods*. Rats were randomly assigned into five groups: control, HS-1200, HCC, HCC + low dose HS-1200, and HCC + high dose HS-1200 groups. Rat HCC model was established by intraperitoneal injection of DEN. And rats were given HS-1200 by daily oral gavage. After 20 weeks, we examined animal body weight, liver weight, liver pathological changes, serum levels of AST, ALT, and AFP, and* mutT homologue gene 1 (MTH1)* in liver tissue.* Results*. Oral gavage of HS-1200 significantly increased animal body weight and decreased liver weight as well as liver coefficient in HCC rats (*P* < 0.05 versus HCC group). Moreover, oral administration of HS-1200 suppressed tumorigenesis, attenuated pathological changes in liver tissues, and decreased serum levels of AST, ALT, and AFP in HCC rats (*P* < 0.05 versus HCC group). In addition, the mRNA level of MTH1 was upregulated in the liver tissues of HCC rats (*P* < 0.05 versus control group), which was reversed by HS-1200 treatment in a dose-dependent manner (*P* < 0.05 versus HCC group).* Conclusions*. HS-1200 inhibits hepatocarcinogenesis and improves liver function maybe by inducing downregulation of MTH1.

## 1. Introduction

Liver cancer is one of the most common malignant tumors and the leading cause of cancer deaths worldwide. An estimated 782,500 new cases of liver cancer and 745,500 liver cancer deaths occurred worldwide in year 2012 [1]. Hepatocellular carcinoma (HCC), which accounts for a majority of primary liver cancers, is generally associated with chronic liver diseases such as hepatitis and cirrhosis [2]. Despite considerable advances in the diagnosis and treatment of primary HCC, the associated mortality rate is still very high due to high metastasis and recurrence rates. At present, the number of drugs used for HCC prevention is quite limited and many of the agents are still undergoing clinical trials [3]. Development of novel treatment options for liver cancer is a key imperative.

Accumulated evidence suggests an anticarcinogenic effect of hydrophilic bile acids such as ursodeoxycholic acid (UDCA). In in vitro cultured HCC cells and in diethylnitrosamine- (DEN-) induced rat HCC model, administration of UDCA appeared to suppress hepatocarcinogenesis by inhibiting cancer cell proliferation [4]. Further, treatment with UDCA was shown to reduce the risk of liver cancer in patients with hepatitis C virus-associated liver cirrhosis [5] and to improve patient prognosis in primary biliary cirrhosis [6]. Chenodeoxycholic acid (CDCA) is the most abundant primary bile acid that is known to decrease dietary cholesterol absorption [7]. HS-1200 {N-[(3a,5b,7a)-3,7-dihydroxy-24-oxocholan-24-yl] b-alanine benzyl ester}, a synthetic CDCA derivative, has shown anticancer activity in several human cancers. For instance, HS-1200 was shown to sensitize breast cancer MCF-7 cell to radiation-induced apoptosis [8]. Further, HS-1200 was shown to inhibit proliferation and promote apoptosis of both prostate cancer cells [9] and bone sarcoma cells [10]. Similarly, proapoptotic effect of HS-1200 on human HCC cell lines BEL7402 and HepG2 cells has also been demonstrated [11, 12]. However, the potential in vivo anticancer effect of HS-1200 against HCC has not been elucidated.

Although the molecular mechanism of HCC development remains largely unknown, oxidative DNA damage is thought to be involved in human hepatocarcinogenesis and progression [13, 14]. Oxidative stress due to excessive production of free radicals such as reactive oxygen species (ROS) is thought to induce genetic instability during carcinogenesis [13]. 8-Hydroxy-2′-deoxyguanosine (8-OHdG) is a well-accepted biomarker of ROS-triggered oxidative DNA damage [15] and is also identified as a risk factor for developing HCC in patients with chronic hepatitis C virus infection [16].* mutT homologue gene 1 (MTH1)*, a hepatocyte DNA repair gene, encodes MTH1 protein which favors the repair of oxidation-induced DNA damage by inhibiting the incorporation of oxidized dNTPs [17, 18]. Oxidative stress upregulates expression of MTH1 which leads to removal of excess of 8-OHdG [19]. Increased expression of MTH1 has been documented in several human cancers such as kidney [20], breast [21], and colorectal cancers [22]. Moreover, increased expression of MTH1 in cancerous tissue as compared to that in adjacent noncancerous tissues has also been demonstrated [23]. However, its precise role in hepatocarcinogenesis is not well understood. We sought to investigate the potential ability of HS-1200 in inhibiting hepatocarcinogenesis and examine the involvement of MTH1 in carcinogenesis. For this purpose, a rat HCC model was established by injecting a chemical carcinogen DEN, which induces liver carcinogenesis by disrupting the antioxidant defense pathway [24]. Our findings indicate that HS-1200 suppressed tumorigenesis and improved liver function in DEN-exposed rats. In addition, the antitumor activity of HS-1200 appears to be associated with its ability to downregulate the mRNA level of MTH1. Our findings suggest a potential role of HS-1200 in reducing oxidative DNA damage and, thereby, preventing HCC.

## 2. Materials and Methods

### 2.1. Animals

A total of 125 eight-week-old male Wistar rats, weighing 180 ± 10 g, were purchased from Animal Experiment Center of Shandong University. Animals were housed adaptively in the center of the Shandong Provincial Hospital Affiliated to Shandong University for 2 weeks. Fifty rats were used for determining the potential hepatotoxicity and nephrotoxicity of HS-1200 treatment. Others were used for the following experiments.

### 2.2. Evaluation of the Hepatotoxicity and Nephrotoxicity of HS-1200

HS-1200 was provided by College of Chemistry, Shandong Normal University, China. To investigate the potential hepatotoxicity and nephrotoxicity of HS-1200, 50 rats were randomly assigned to five groups based on the dosage of HS-1200: control (*N* = 10), 20 mg/kg HS-1200 (*N* = 10), 40 mg/kg HS-1200 (*N* = 10), 80 mg/kg HS-1200 (*N* = 10), and 100 mg/kg HS-1200 (*N* = 10) group. In HS-1200 groups, animals received daily intragastric administration of different doses of HS-1200 for 40 weeks. Control rats received daily intragastric administration of normal saline solution for the same period. Tail venous blood was collected from each animal for biochemical analysis of liver and renal function every two weeks. Serum levels of alanine aminotransferase (ALT), aspartate aminotransferase (AST), gamma-glutamyl transpeptidase (GGT), albumin (ALB), urea nitrogen (BUN), and creatinine (Cr) were determined using ELISA kits (Shanghai Enzyme-Linked Biotechnology Co., Ltd., China), as per the manufacturer's instructions. After 40 weeks, animals were sacrificed and the histological changes in liver and kidney examined under light (Olympus, Japan) and electron microscope (JEM-100S, Hitachi, Japan).

### 2.3. Establishment of Rat Model of Primary Hepatic Carcinoma

Primary hepatic carcinoma model of rat was established by intraperitoneal injection of 2 g/L diethylnitrosamine (DEN, Sigma, USA), twice a week for six consecutive weeks. The dose of DEN was initiated from 20 mg/kg and gradually increased until 60 mg/kg (20, 20, 40, 40, 60, and 60 mg/kg). The animals were routinely housed and fed for a total of 20 weeks. No animal deaths occurred during the experiment.

### 2.4. Experimental Assignment

After 2 weeks of adaptive feeding, rats were randomly assigned to five groups: control (*N* = 15), HS-1200 (*N* = 15), HCC (*N* = 15), HCC + low dose HS-1200 (*N* = 15), and HCC + high dose HS-1200 (*N* = 15) groups. Primary rat HCC model was established as described above. In HCC + low dose HS-1200 or HCC + high dose HS-1200 groups, rats were given HS-1200 by daily oral gavage at a dose of 40 and 80 mg/kg, respectively, for 18 weeks (from 3rd week to 20th week). Control rats received equivalent intraperitoneal injection of normal saline solution for the first six weeks without daily oral gavage of HS-1200. Rats in HS-1200 group were given equivalent intraperitoneal injection of normal saline for the first six weeks and daily oral gavage of 80 mg/kg HS-1200 for 18 weeks (from 3rd week to 20th week). Drugs were administered at the same time every day. Animal body weights were measured at the end of the 20th week.

### 2.5. Pathological Examination

Rats were sacrificed at the end of the 20th week. The liver was carefully removed and weighed. Liver coefficient was calculated using the following equation:

(1) $$\begin{array}{c} Liver\;\;coefficient\;\;\left(\;\%\;\right)=\frac{Liver\;\;weight\;\;\left(g\right)}{Body\;\;weight\;\;\left(g\right)}. \end{array}$$

Liver samples were fixed and embedded in paraffin. Hematoxylin and eosin (H&E) stained sections were examined under microscope (Olympus, Japan) at a magnification of 200x.

### 2.6. Serological Analysis

Before sacrificing the animals, 2 mL blood samples were drawn by performing cardiac puncture. Serum levels of ALT, AST, and *α*-fetoprotein (AFP) were determined using ELISA kits (Shanghai Enzyme-Linked Biotechnology Co., Ltd., China), as per the manufacturer's instructions.

### 2.7. Real-Time Quantitative RT-PCR

Total RNA was extracted from liver tissue specimens obtained from each group using Trizol reagent (TaKaRa Biotechnology Co., Ltd., Japan). RNA was reverse transcribed into cDNA using PrimeScript™RT reagent kit (TaKaRa Biotechnology Co., Ltd., Japan). For PCR analysis, real-time PCR amplification kit (SYBR Premix Ex Taq™, TaKaRa Biotechnology Co., Ltd., Japan) was used. A rat *β*-actin Housekeeping Gene Primer Set (TaKaRa Biotechnology Co., Ltd., Japan) was used as control. The mRNA expression of MTH1 was examined by real-time quantitative RT-PCR; results were normalized to mRNA levels of *β*-actin.

### 2.8. Statistical Analysis

Statistical analyses were performed using SPSS 15.0 software (SPSS Co., USA). Data are presented as mean ± SD. Data expressed as frequencies were analyzed by Chi-squared test. Variables with normal distribution were compared by single factor Analysis of Variance with LSD analysis. Intergroup differences with associated *P* values of <0.05 were considered statistically significant.

## 3. Results

### 3.1. Toxicity Assessment of HS-1200

**Figure 1:**
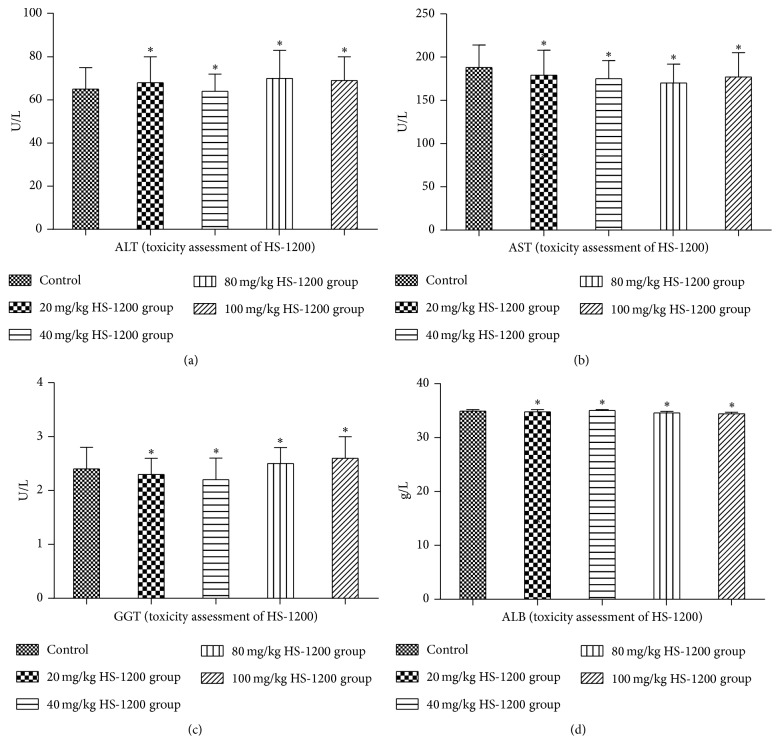
Liver function of HS-1200 toxicity assessment groups. (a) Serum ALT; (b) serum AST; (c) serum GGT; (d) serum ALB. Data expressed as mean ± SD. ^*∗*^*P* > 0.05 versus control. ALT, alanine aminotransferase; AST, aspartate aminotransferase; GGT, transpeptidase; ALB, albumin. HS-1200, N-[(3a,5b,7a)-3,7-dihydroxy-24-oxocholan-24-yl] b-alanine benzyl ester.

**Figure 2:**
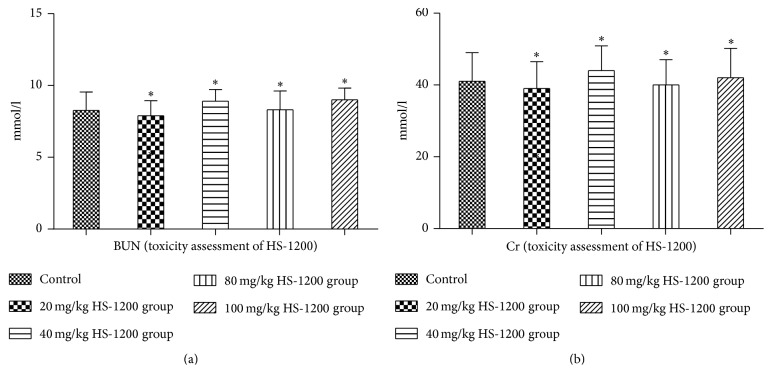
Renal function of HS-1200 toxicity assessment groups. (a) Serum BUN; (b) serum Cr. Data expressed as mean ± SD. ^*∗*^*P* > 0.05 versus control. BUN, urea nitrogen; Cr, creatinine. HS-1200, N-[(3a,5b,7a)-3,7-dihydroxy-24-oxocholan-24-yl] b-alanine benzyl ester.

We assessed the potential hepatotoxicity and nephrotoxicity of HS-1200 treatment in rats. For this purpose, animals received intragastric HS-1200 (daily dose ranging from 0 to 100 mg/kg). Liver function (ALT, AST, GGT, and ALB) (Figure 1) and renal function (BUN, Cr) (Figure 2) were determined in each rats group at all time points. All test results are within the normal range. No significant difference was observed in the liver function and renal function of any of the HS-1200 toxicity assessment groups (*P* > 0.05 versus control; Figures 1 and 2). After 40 weeks of uninterrupted treatment, pathological examinations were all normal, not necessary to be shown here. So no significant hepatotoxicity or nephrotoxicity was detected in any of the groups. The results suggested that HS-1200 was relatively safe for rats in the dose range of 0 to 100 mg/kg/d. And the dose of 40 and 80 mg/kg/d was decided for treating the rats as low dose and high dose in the next experiment.

### 3.2. HS-1200 Treatment Inhibits Hepatocarcinogenesis

**Figure 3:**
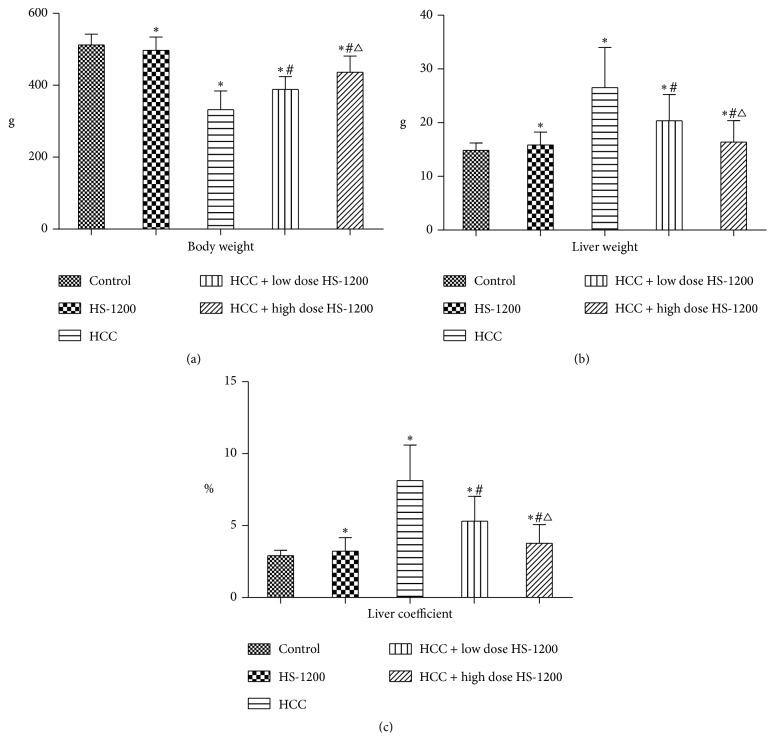
Body weight, liver weight, and liver coefficient of study groups. (a) Animal body weight; (b) liver weight; (c) liver coefficient at the end of 20th week. Data expressed as mean ± SD. ^*∗*^*P* < 0.05 versus control; ^#^*P* < 0.05 versus HCC; ^△^*P* < 0.05 versus HCC + low dose HS-1200. HCC, hepatocellular carcinoma; HS-1200, N-[(3a,5b,7a)-3,7-dihydroxy-24-oxocholan-24-yl] b-alanine benzyl ester.

**Figure 4:**
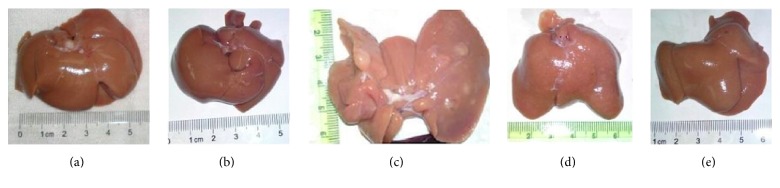
Gross liver examination: (a) control; (b) HS-1200; (c) HCC; (d) HCC + low dose HS-1200; (e) HCC + high dose HS-1200. HCC, hepatocellular carcinoma; HS-1200, N-[(3a,5b,7a)-3,7-dihydroxy-24-oxocholan-24-yl] b-alanine benzyl ester.

After treatment with DEN, primary HCC was induced in 93% (14/15) rats. HCC rats exhibited a significant loss of body weight as compared to that in the control group (control, 512 ± 30 g; HCC, 332 ± 52 g; *P* < 0.05; Figure 3(a)). This was accompanied by significantly greater liver weight and liver coefficient in the HCC rats (*P* < 0.05 versus control; Figures 3(b) and 3(c)). Low dose and high dose HS-1200 treatment remarkably reduced tumorigenesis by 80% (12/15) and 60% (9/15), respectively. When compared to HCC group, animals receiving low dose or high dose HS-1200 therapy showed increased body weight, reduced liver weight, and decreased liver coefficient (*P* < 0.05 versus HCC; Figure 1). Moreover, high dose HS-1200 treatment appeared to have a greater efficacy in inhibiting hepatocarcinogenesis (Figure 3). Gross liver examination revealed obvious tumor masses in HCC rats, while in the low dose or high dose HS-1200 groups, tumor growth was suppressed (Figure 4).

### 3.3. HS-1200 Treatment Attenuated DEN-Induced Pathological Changes in Liver

**Figure 5:**
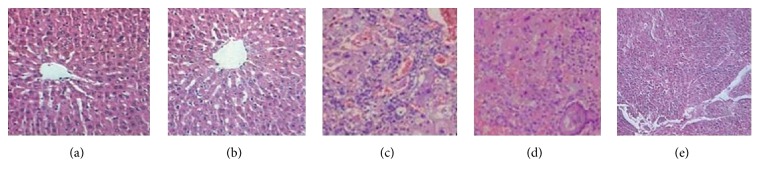
Histopathological examination of liver tissues. H&E stained sections of liver specimens were examined under microscope at 200x magnification. (a) Control; (b) HS-1200; (c) HCC; (d) HCC + low dose HS-1200; (e) HCC + high dose HS-1200. H&E, hematoxylin and eosin; HCC, hepatocellular carcinoma; HS-1200, N-[(3a,5b,7a)-3,7-dihydroxy-24-oxocholan-24-yl] b-alanine benzyl ester.

We next compared the histological findings in liver among the five experimental groups. In the liver tissues obtained from the control or HS-1200 groups, hepatocytes were arranged around the central vein in a radial configuration, and hepatic lobule structures were well preserved (Figures 5(a) and 5(b)). In contrast, disorganized hepatocytes and hepatic lobules, pseudolobule formation, cancer cell nests, and tumor cell atypia were observed in livers of HCC rats (Figure 5(c)). In addition, HCC rats exhibited hepatocyte necrosis as well as severe hepatic inflammatory cell infiltration. In HCC rats receiving low dose of HS-1200 treatment, the cancer cell nest formation and cell necrosis appeared to be much attenuated (Figure 5(d)). High dose of HS-1200 therapy was associated with much improved pathological findings in HCC rats as compared to that observed in rats in the low dose HS-1200 treatment group (Figure 5(e)).

### 3.4. HS-1200 Prevents Liver Dysfunction in HCC Rats

**Figure 6:**
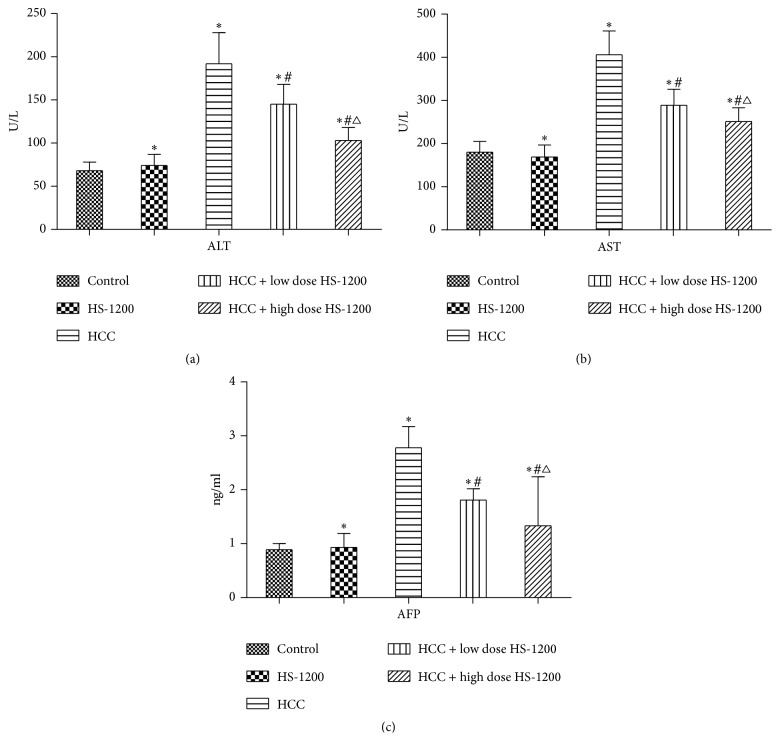
Serum ALT, AST, and AFP levels of study groups. (a) Serum ALT; (b) serum AST; (c) AFP. Data expressed as mean ± SD. ^*∗*^*P* < 0.05 versus control; ^#^*P* < 0.05 versus HCC; ^△^*P* < 0.05 versus HCC + low dose HS-1200. ALT, alanine aminotransferase; AST, aspartate aminotransferase; AFP, Alpha-fetoprotein. HS-1200, N-[(3a,5b,7a)-3,7-dihydroxy-24-oxocholan-24-yl] b-alanine benzyl ester.

To evaluate the impact of HS-1200 therapy on liver function, serum levels of ALT, AST, and AFP in rats in the different experimental groups were compared. Serum levels of all three biochemical parameters were significantly elevated in HCC rats (*P* < 0.05 versus control), which indicated liver dysfunction in these animals (Figure 6). Administration of low or high dose of HS-1200 appeared to reverse the upregulation of serum ALT, AST, and AFP expressions in HCC rats (*P* < 0.05 versus HCC). Moreover, the serum levels of these parameters were lower in the HCC rats in the high dose HS-1200 group (*P* < 0.05 versus HCC + low dose HS-1200).

### 3.5. HS-1200 Downregulates Hepatic mRNA Expression of MTH1 in HCC Rats

**Figure 7:**
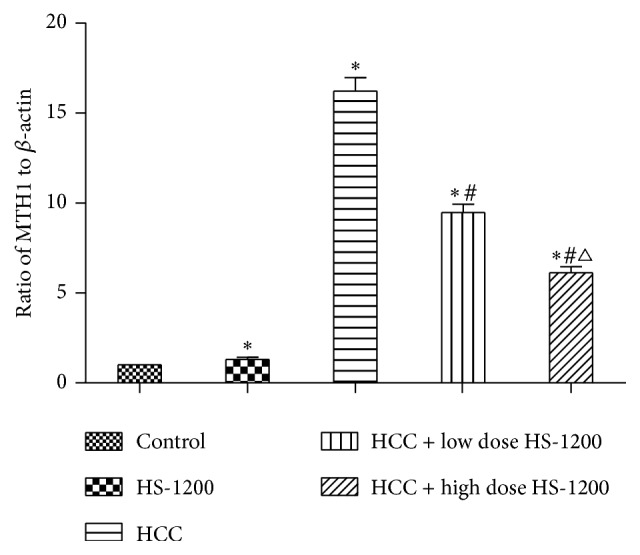
Results of real-time quantitative PCR for mRNA expression of MTH1 in liver tissues. mRNA levels of MTH1 are normalized to *β*-actin levels. ^*∗*^*P* < 0.05 versus control; ^#^*P* < 0.05 versus HCC; ^△^*P* < 0.05 versus HCC + low dose HS-1200. PCR, polymerase chain reaction; MTH1, mutT homologue gene 1; HCC, hepatocellular carcinoma; HS-1200, N-[(3a,5b,7a)-3,7-dihydroxy-24-oxocholan-24-yl] b-alanine benzyl ester.

To investigate the molecular mechanisms involved in HS-1200-mediated inhibition of tumorigenesis, we examined the mRNA expression of MTH1 in liver tissues. We found significant upregulation of mRNA level of MTH1 in liver tissues of HCC rats (control, 1; HCC, 16.23 ± 0.74; *P* < 0.05; Figure 7). Further, upregulation of MTH1 mRNA was significantly reversed by low or high dose HS-1200 therapy; high dose appeared to have a higher efficacy in this respect (HCC + low dose HS-1200, 9.48 ± 0.46; HCC + high dose HS-1200, 6.13 ± 0.33; *P* < 0.05 versus HCC).

## 4. Discussion

In the present study, a rat primary HCC model was successfully established by DEN injection. Oral administration of HS-1200 significantly attenuated tumorigenesis and pathological changes in liver tissues, as well as improving liver function of rats exposed to DEN. In addition, the inhibitory role of HS-1200 against HCC positively correlated with the dose of HS-1200.

Although bile acids have been shown to be causative agents in several cancers of the digestive system, including stomach, biliary tract, and colon [25], the precise effect of bile acids on hepatocarcinogenesis is largely unclear. Cholestasis, associated with excessive production of bile acids, is a known etiological factor for liver injury [26]. However, hydrophilic bile acids such as UDCA and CDCA differ in the physicochemical and biological characteristics, and hepatoprotective properties of these subgroups of bile acids have been reported [25]. Liang et al. reported that the CDCA-verticinone ester caused cell cycle arrest, induced ROS generation, and induced cell apoptosis in cultured human liver carcinoma HepG2 cells [27]. The cytotoxic effects of bile acid derivatives, including HS-1183, HS-1199, and HS-1200, have also been demonstrated in in vitro cultured human breast carcinoma cells [28].

In the present study, daily oral gavage of HS-1200 (80 mg/kg) had no significant toxic effect on liver or renal function in rats. However, potential toxicity of HS-1200 at doses over 100 mg/kg/d was not evaluated. Moreover, we demonstrated for the first time the antihepatocarcinogenesis activity of HS-1200 in a primary HCC rat model. Oral gavage of HS-1200 at both low (40 mg/kg/d) and high (80 mg/kg/d) dose inhibited tumorigenesis in DEN-exposed rats. These findings are consistent with recent reports which showed that administration of UDCA blocks the in vivo growth and induce apoptosis of BEL7402 cells in mice [29]. In addition, HS-1200 appeared to prevent pathological changes in liver and reversed the elevated serum levels of ALT, AST, and AFP in rats exposed to DEN. The hepatoprotective effect of HS-1200 appears to be dose-dependent as indicated by our results.

By now, we have not found any side effects of HS-1200 in any literature, and no significant hepatotoxicity or nephrotoxicity was detected in any of our experimental groups. A few of the rats with HS-1200 administration suffered from mild diarrhea in the process of experiment. But the diarrhea rats had no obvious mental state change and can tolerate the diarrhea. It might suggest that diarrhea be a potential side effect of HS-1200. We will continue to study it in next research.

DNA damage is very important in the development and progression of liver cancer [30], and MTH1 plays an important role in the repair of DNA oxidative damage. As we mentioned in introduction, 8-OHdG is commonly used biomarker in DNA damage [15]. MTH1's gene encoding production is a kind of triphosphatase, with a glycosidic enzyme activity. It can hydrolyze 8-OH-dGTP into 8-OH-dGMP in free nucleotides pool and so inhibit 8-OHdG incorporating to DNA chain by mistake, reduce the occurrence of DNA mutations, and then avoid the damage of DNA [31]. Studies have documented that activation of a DNA repair gene MTH1 is required for cancer cells to block incorporation of oxidized dNTPs and thereby prevent ROS-induced DNA damage [18, 32]. MTH1 expression can be increased to eliminate the excess of 8-OHdG produced in oxidative stress [19]. Upregulation of MTH1 has been demonstrated in several types of cancer cells, including in HCC [20–22]. According to the study of Obtułowicz et al., malignant colorectal cancer could cause oxidative stress of tumor cells and promote the upregulation of MTH1 [33]. Borrego et al. found expression level of MTH1 had positive correlation with malignant degree of gastric carcinoma cells [34]. Kennedy et al. indicated that MTH1 was overexpressed in lung cancer cells. And they pointed out MTH1 was necessary for lung cancer cells to remove the damaged DNA structure and maintain the division of cancer cells properly [35]. Jüngst et al. found that MTH1 expression was significantly higher in liver tumor than corresponding nontumor tissues [23]. MH1 can be as an index to reflect DNA repair and ability of oxidative stress of liver cell [36]. Nakabeppu also reported MTH1 inhibitor could increase oxidative damage, increase the cytotoxicity, and induce apoptosis in cancer cells. And he showed MTH1 inhibitor could be a better strategy for treatment of targeting therapy for cancer [37].

Consistent with the current body of evidence, we documented an increased expression of MTH1 mRNA in the liver tissues of rats exposed to DEN. Further, administration of HS-1200 appeared to reverse the upregulation of MTH1 mRNA in a dose-dependent manner. Our findings suggest that HS-1200 may prevent tumorigenesis and protect against liver damage by inducing downregulation of MTH1 and facilitate DNA repair in liver tissues. Indeed, development of small-molecule inhibitors targeting MTH1 has been suggested as a promising strategy for anticancer treatment [18, 38]. However, another issue should also be considered. It has shown MTH1-deficient mice tended to develop liver tumors spontaneously [39] in other studies. Hence, the endogenous level of MTH1 if maintained at a balanced level may minimize tumorigenesis.

In this study, HS-1200 inhibited hepatocarcinogenesis and improved liver function in HCC rats possibly by downregulation of MTH1 and enhanced DNA repair. The hepatoprotective effect of HS-1200 showed a positive correlation with its dose. We tentatively suggest that the mechanism may be through the P53 signaling pathways, and future studies are required to further explore the underlying mechanisms that mediate the antitumor and hepatoprotective effect of HS-1200. HS-1200 has earlier been shown to be apoptosis inducer, inhibit cell proliferation, and also be inducer of autophagic cell death in various studies. Along with various other mechanisms, HS-1200 might act through downregulating MTH1 gene as observed in our study.
